# Author Correction: PND-Net: plant nutrition deficiency and disease classification using graph convolutional network

**DOI:** 10.1038/s41598-024-68260-7

**Published:** 2024-08-05

**Authors:** Asish Bera, Debotosh Bhattacharjee, Ondrej Krejcar

**Affiliations:** 1 Department of Computer Science and Information Systems, BITS Pilani, Pilani Campus, Pilani, Rajasthan 333031 India; 2 Department of Computer Science and Engineering, Jadavpur University, Kolkata, West Bengal 700032 India; 3 Faculty of Informatics and Management, University of Hradec Kralove, Hradec Kralove, Czech Republic; 4 Skoda Auto University, Na Karmeli 1457, 293 01 Mlada Boleslav, Czech Republic; 5 Malaysia Japan International Institute of Technology (MJIIT), Universiti Teknologi Malaysia, Kuala Lumpur, Malaysia

Correction to: *Scientific Reports* 10.1038/s41598-024-66543-7, published online 05 July 2024

The Acknowledgements section in the original version of this Article was incomplete.

“This work is supported by the New Faculty Seed Grant (NFSG) and Cross-Disciplinary Research Framework (CDRF: C1/23/168) projects, Open Access facilities, and necessary computational infrastructure at the Birla Institute of Technology and Science (BITS) Pilani, Pilani Campus, Rajasthan, 333031, India.”

now reads:

“This work is supported by the New Faculty Seed Grant (NFSG) and Cross-Disciplinary Research Framework (CDRF: C1/23/168) projects, Open Access facilities, and necessary computational infrastructure at the Birla Institute of Technology and Science (BITS) Pilani, Pilani Campus, Rajasthan, 333031, India. This work has been also supported in part by the project (2024/2204), Grant Agency of Excellence, University of Hradec Kralove, Faculty of Informatics and Management, Czech Republic.”

The original Article has been corrected.

